# A Sketch of the Weather at Londonderry, in the Year 1801

**Published:** 1802-06

**Authors:** 

**Affiliations:** that Place


### *A Sketch of the Weather at Londonderry, in the Year* 1801; *communicated by Dr.* PATTERSON, *of that Place.*

THE commencement of *January* was boisterous, but the weather soon grew mild and genial; which temperature, with little exception, it preserved throughout the month; and some of the nights, particularly at the beginning, were softer and warmer than the preceding days. There were, indeed, several fresh and cool breezes, together with some sharp, but short, congelation, and three or four days of foggy air. The prevalent winds were W. and S. W. The maximum of Fahrenheit's thermometer was 46½°; its minimum, 36°. In *February* the winds were generally moderate; the heat of the air was mostly temperate, sometimes tepid; and this state of the air air often prevailed in the night as well as in the day. The blowing and wet weather that did occur, took place more during the nights than the days. In thefe two months, the warm winds were to the cold in the ratio of 55 to 28. In this month, thermometer, max. 52°; min. 35°. *March* produced frequent frefh breezes, and chiefly from the warmer points; often fhowers alfo, and fome heavy rain; both wind and rain were greater in the courfe of the nights than of the days; and the temperature of the air, which was cold till the 24th, then changed to fo warm a ftate, that perfons accuftomed to wear great coats laid them afide; no wonder, when the thermometer rofe to 61° in the fhade; its minimum was 36°. Prevalent winds W. and S. W. Though there was a good deal of hail and fome fnow in *April*, yet the ground was fo warm that they foon vanifhed; and the general temperature of the month was remarkably pleafant and genial; and in the latter part of the month, there being good moonlight, the nights as well as the days were quite delightful. Prevalent winds were S. and W. Thermometer, max. 71; min. 33°. The beginning of *May* was equally dry with *April*; and between the two months there were twenty-four days, during which hardly any rain fell. The force of the winds was partly ftrong, partly moderate; and their direction generally came from the colder quarters, which made this month lefs genial than the preceding. Thermometer, max. 67½°; min. 49°. The winds in *June* were generally moderate in force, but proceeded from cold points, principally N.; though the fky was often covered with clouds, and indicated both rain and thunder, yet none of the latter, and comparatively little of the former, happened; whilft a confiderable degree of exficcation took place in the air. Thermometer, max. 72°; min. 53°. In *July* the winds were more frequently moderate than otherwife; a good deal of rain fell, and both wind and rain were greater in the nights than in the days. The fky was often extenfively covered with common clouds, and feveral times it was ftreaked with plumous ones, whilft the air was frequently fultry, although the atmofphere was fo charged with cloudy vapours. Prevalent winds were W. and S. W. Thermometer, max. 71½°; min. 53°. The winds in *Auguft* were generally moderate, often eafy, and but few frefh breezes occurred. The air was fometimes foggy, at other times a hazinefs appeared high in the atmofphere; again the fky was covered and lowering, yet frequently the fun fhone bright. The temperature of the air for the moft part was warm; in turns it was fultry; and taking the month in general, the weather was remarkably fine. In the evening and night of the 12th and 13th days of this month, a confiderable quantity of vivid, but filent,

ſilent, lightning filled the atmoſphere The predominant direction of the winds was divided between S. W. and N. E. Thermometer, max. 76°; min. 57°.

Some blowing weather occurred in *September*, but in general the wind was moderate; the ſky was now and then overcaſt, yet commonly clear. For the moſt part the air was warm; and, two or three days, it poſſeſſed a degree of heat which, to the ſenſations, ſeemed as if it iſſued out of the mouth of a hot oven; at this time the thermometer roſe in the ſhade, two o'clock, p. m. no higher than 67°; its max. throughout the month was 69½°; min. 52°. In *October* there was frequent blowing weather; ſometimes the gales were conſiderable, with heavy ſhowers, and both wind and rain were greateſt in the nights, very early in the mornings, and late in the evenings. Some days, the air had a pleaſant warmth, which twice or thrice extended to the beginning of night. The W. and S. W. winds were predominant. Thermometer, max. 57°; min. 40°. Squally gales, ſometimes ſtormy ones, proceeding chiefly from N. W. occurred in *November*; a conſiderable quantity of hail fell, and ſome ſnow, which together whitened both the low grounds and mountains; yet, in the middle of the month, the temperature of the air was comparatively mild, and the beginning of ſome nights was warmer than the preceding days. The firſt congelation, in the decline of this year, happened the 2d of this month, and the firſt ſnow on the 4th. The prevailing winds were W. and N. W. Thermometer, max. 53°; min. 33°. Froſt occurred often, and the degree of congelation ſometimes was conſiderable in *December*, but it ſuffered frequent interruptions. Snow alſo fell ſeveral times, but upon the whole it did not accumulate more than four or five inches in depth from the ſurface of the ground, and, like the froſt, its continuance was broken by intervals of rain. The winds, which were partly boiſterous, partly moderate, were, with the rains, greateſt during the nights. The cold winds were to the warm, as 27 to 15. Thermometer, max. 42°; min. 22°.

